# A comparison of the effects of Methylprednisolone Acetate, Sodium Hyaluronate and Tenoxicam in the treatment of non-reducing disc displacement of the temporomandibular joint

**DOI:** 10.4317/medoral.22237

**Published:** 2018-04-24

**Authors:** Günay Yapıcı-Yavuz, Göksel Şimşek-Kaya, Hayri Oğul

**Affiliations:** 1 Department of Oral and Maxillofacial Surgery, Faculty of Dentistry, Adıyaman University, Adıyaman, Turkey; 2 Department of Oral and Maxillofacial Surgery, Faculty of Dentistry, Akdeniz University, Antalya, Turkey; 3 Department of Radiology, Faculty of Medicine, Atatürk University, Erzurum, Turkey

## Abstract

**Background:**

This clinical study aimed to radiologically and clinically compare the effect of intra-articular injection of methylprednisolone, sodium hyaluronate or tenoxicam following arthrocentesis with that of arthrocentesis alone in patients with non-reducing disc displacement.

**Material and Methods:**

A total of 44 patients radiographically diagnosed with non-reducing disc displacement of the temporomandibular joint (TMJ) were randomly divided into four treatment groups, as follows: Group 1, arthrocentesis alone; Group 2, arthrocentesis plus methylprednisolone acetate; Group 3, arthrocentesis plus sodium hyaluronate; Group 4, arthrocentesis plus tenoxicam. Maximum mouth opening (MMO), lateral movement, pain severity and tenderness of TMJ and muscles of mastication on palpation were measured before treatment and at 1 week and 1, 3 and 6 months after treatment. Disc position, presence or absence of disc reduction, level of effusion, joint movement and joint space were also evaluated using magnetic resonance imaging (MRI) before treatment and 6 months after treatment.

**Results:**

No significant differences in treatment success were found among the four groups. MRI findings did not vary significantly among the groups, but pre- and post-operative MRI findings varied significantly within all four groups (*p*<0.001).

**Conclusions:**

According to the data from this study, it may be concluded that either arthrocentesis alone or arthrocentesis with methylprednisolone acetate or sodium hyaluronate or tenoxicam intra-articular injections are similarly effective and promising methods in the treatment of TMJ with non-reducing disc displacement.

** Key words:**Non-reduction disc displacement, arthrocentesis, methylprednisolone, sodium hyaluronate, tenoxicam.

## Introduction

Arthrocentesis, i.e. lavage of the superior joint space, was first described by Nitzan in 1991 (1). The main indications for arthrocentesis are acute or chronic restricted movement deriving from non-reducing disc displacement and hypomobility associated with limited condyle translation in the superior joint space (2). Arthrocentesis may also be indicated for patients with osteoarthritis and rheumatoid arthritis. Arthrocentesis reduces pain by eliminating inflammatory mediators (3), increases condyle mobility by eliminating intra-articular negative pressure and adhesions (1,3), and increases the mobility of a joint whose movement has been constricted as a result of anterior displacement (3). Arthrocentesis is a simple procedure that can be repeated if necessary, requires no special equipment, and has low complication rates, making it a popular and widely used technique in the treatment of internal derangements of the temporomandibular joint (TMJ) (3).

Intra-articular drug injections can be applied alone or following arthrocentesis or arthroscopic surgery; however, the effectiveness of intra-articular drug injections following arthrocentesis is still controversial (4). Previous studies have looked at the intra-articular application of various materials, including glucocorticosteroids, sodium hyaluronate (SH), tenoxicam, morphine and bupivacaine (1,5-8).

Some authors have reported intra-articular corticosteroid injections to reduce pain and improve function (8). SH, which has a high molecular weight and high viscosity, has also been shown to reduce friction and pain and improve joint mobility by restoring soft-tissue lubrication; to play role in anti-inflammation and buffering, and to repair articular cartilage nutrition (9). The decrease in viscoelasticity and molecular weight of hyaluronate decrease in joints exposed to degenerative changes, thereby increasingly disposing them to cartilage injury. It has been suggested that exogenous administration of hyaluronate stimulates hyaluronate production by synoviocytes inside the joint, reducing friction and thus protecting articular structures (10). It has also been suggested that the biochemical structure of the joint can be restored to normal by replacing low-molecular-weight hyaluronate in the inflamed joint with high-molecular weight-hyaluronate applied exogenously (9).

A derivative of oxicam that falls within the enolic-acid group of non-steroidal anti-inflammatory drugs (NSAID), tenoxicam has exhibited both anti-inflammatory and analgesic properties. Intra-articular application has been found to be more effective than oral and intravenous application in terms of analgesia (11); however, very few studies have investigated administration of tenoxicam into the TMJ (6,7).

Given the lack of consensus on drug use after arthrocentesis, additional clinical studies are required. To the best of our knowledge, no previous study in the English-language literature has compared the effectiveness of SH, dexamethasone and tenoxicam in the treatment of non-reducing disc displacement of the TMJ. Therefore, this study radiologically and clinically compared the effects of SH, dexamethasone and tenoxicam injected intra-articularly following arthrocentesis with the effects of arthrocentesis alone in patients with non-reducing disc displacement.

## Material and Methods

- Patients

This prospective, randomized, single-blinded study was conducted at the Atatürk University Faculty of Dentistry’s Department of Oral and Maxillofacial Surgery between May 2011 and April 2013. The study was conducted in line with the principles of the 1975 Helsinki Declaration for biomedical research involving human subjects, as revised in 2004, and was approved by the Atatürk University Human Research Ethics Committee of Faculty of Dentistry under protocol 2011/008 and Atatürk University Health Sciences Institutional Review Board for Human Studies under protocol 2012/2012.2.12. Patients were informed about the surgery, postoperative recommendations and possible complications before the procedure, and all participants gave their written consent.

The patients who were referred to our clinic with complaints of TMJ pain, TMJ noises, and limitation of mouth opening were examined clinically and with magnetic resonance images. Inclusion criteria were: Increased TMJ pain on chewing or during maximal mouth opening; TMJ pain of at least 3 on a 0–10 visual analogue scale (VAS) (rated from 0=no pain to 10=worst pain imaginable using a visual analogue scale); a history of joint clicking and limited mouth opening (<35 mm), together with a deviation to the affected side and an increase of up to 3 mm in mouth opening with assistance; limited lateral and protrusive movement, with deviation to the affected side; symptoms duration of at least 2 months; a primary TMJ pain complaint diagnosed as arthralgia and disc displacement without reduction according to the Research Diagnostic Criteria (RDC)/TMD (12). In all patients, the presence of non-reducing disc displacement was confirmed by magnetic resonance imaging (MRI). No patient had previously been treated for disorders of the TMJ. Exclusion criteria were as follows: Pregnancy and lactation; presence of a medical condition or drug use that might affect the surgical procedure or post-surgical healing; previous exposure of the TMJ to direct trauma or fracture of facial bones, or previous history of TMJ surgery; signs or symptoms of myalgia; condylar hypoplasia/hyperplasia or tumor, presence of facial growth disorder or systemic inflammatory joint disease, or history of severe degenerative joint disease (MRI signs of deformed condylar contour or osteopenia together with osteophyte or roughness of the condylar surface and bony changes in the glenoid fossa) or bony/fibrous adhesion; or contraindication at MRI. Patient psychological status was also evaluated at the start of treatment, and patients with depression or somatization according to Dworkin and Le Resche (13) were also excluded.

52 individuals were initially selected but of these, 8 were excluded according to the exclusion criteria. Sample selection was discontinued at 44 patients (38 wo-men, 6 men). Patient demographics, medical and dental anamneses and TMJ examinations were completed during the initial presentation. Clinical examinations were conducted, and all patient symptoms (including initial symptoms and symptom durations), maximum mouth opening (MMO) and protrusive and lateral movement of the mandible, degree of pain on bimanual palpation of the muscles of mastication and TMJ region (rated from 0=no pain to 10=worst pain imaginable using a VAS) and tooth grinding/clenching habits were recorded.

- Radiological Examination

Clinical diagnosis was confirmed through MRI examinations. MRIs were taken with the mouth open and closed. Disc position, presence or absence of disc reduction, amount of effusion, range of motion and superior joint space width were recorded. Normal disc position was defined based on the location of the posterior band of the disc at the superior position relative to the condyle. Effusion levels were classified according to Larheim *et al.* (14) (0: no effusion; 1: effusion in the form of a dot or line along the articular surfaces, 2: moderate effusion, 3: severe effusion). A diagnosis of an internal derangement classifiable as non-reducing disc displacement was made based on disc displacement with the mouth closed and no interposition of the disc between the condyle and articular eminence with the mouth open (15). Pre- and post-treatment MRIs were independently evaluated by a radiologist blinded to the clinical diagnosis and therapeutic protocol.

- Arthrocentesis 

All surgical procedures were performed by the same surgeon (G.Y.Y.). The patient was seated at a 45° angle on a dental chair with the head turned towards the unaffected side. The target site was prepared, and the external auditory meatus was blocked with damp cotton. The ear and periaruricular region were wiped with antiseptic solution, and areas outside the procedure site were covered with a sterile covering. Local anesthesia (2 ml Ultracain® DS Forte 40 mg/ml articaine HCI, 1.2 µg/ml epinephrine, Sanofi-Aventis İlaçları, Istanbul, Turkey) was administered to block the auriculotemporal nerve, and arthrocentesis was performed according to the technique described by Nitzan *et al.* (1). This procedure was performed for 15-20 min using 200 cc of Ringer’s lactate under adequate pressure in order to eliminate catabolytes present in the synovial fluid. Patients were instructed to continuously open and close their mouths during the procedure in order to restore MMO. Next, one of the needles was removed and, depending on the study group concerned, either methylprednisolone acetate, SH, or tenoxicam was injected using the remaining needle. (In the case of the control group, no drug was administered). Once the procedures were finished, the mandible was gently manipulated along the vertical, protrusive and lateral planes in order to facilitate adhesion lysis and free up the disc still further. No complications were observed during or after the procedures.

In order to prevent any relapse or joint deterioration due to bruxism (16), all patients were provided with stabilization splints, which were prepared according to Okeson (17) and which were used only at night for 6 months following arthrocentesis. Patients were also instructed to perform active and passive mouth-opening exercises and follow a soft diet for one week following treatment procedures. Follow-up appointments were scheduled for 1 week and 1, 3 and 6 months postoperatively. At each follow-up session, occlusal contacts were monitored, and adjustments were made as necessary.

- Treatment groups 

Patients were randomly assigned into one of four treatment groups: Arthrocentesis (Group I); Arthrocentesis + intra-articular SH (Hyalgan®, Bilim İlaç, Istanbul, Turkey) injection (Group II); Arthrocentesis + intra-articular methylprednisolone acetate (Depo-medrol, Pfizer İlaçları, Istanbul, Turkey) injection (Group III); and Arthrocentesis + tenoxicam (Oksamen L, Mustafa Nevzat İlaç Sanayi, Istanbul, Turkey) injection (Group IV).

- Postoperative evaluation 

All patients were evaluated by a surgeon blinded to patient treatment, immediately before arthrocentesis and at 1 week and 1, 3 and 6 months postoperatively, and MMO, VAS pain scores and muscles of mastication and joint palpation values were recorded.

MRIs were obtained from all patients at 6 months post-treatment and used to assess disc position, disc reduction, effusion level, joint range of motion and superior joint space width.

Arthrocentesis treatment success was evaluated at 6 months according to American Association of Oral and Maxillofacial Surgeons criteria (18), as follows: MMO >35 mm; VAS<2 (little or no pain); lateral movement >6 mm.

- Statistical Analysis

Statistical analysis was performed using SAS (Statistical Analysis System Version 13.0 2002, Cary, NC) software. We assessed the assumption of sampling distribution such as normality (controlled with Kolmogorov Smirnov test) before statistical analysis and the hypothesis for normality was met. Descriptive data (means and standard deviations) were calculated, and one-Way ANOVA was used to compare numerical data among groups, while chi-square tests were used to compare qualitative data among groups. McNemar test was utilized to compare pre and post-treatment 6th monthly MRI data. Results were analyzed at a significance level of *p*<0.05.

## Results

- Clinical findings 

No significant complications were reported. No statistically significant differences in mean age or gender distribution were identified among groups (*p*<0.05). Although there were more women than men in all groups, the differences were not statistically significant.

No statistically significant differences among groups were observed in psychological values, duration of symptoms (in months) (*p*<0.05). No statistically significant differences among groups were observed in presence of bruxism, malocclusion, edentulism, or previous history of trauma considered capable of affecting therapeutic outcomes (*p*<0.05).

Neither lateral movement nor MMO varied significantly among the groups (*p*<0.05), ( Table 1). Similarly, decreases in VAS scores did not vary significantly among groups (*p*<0.05), ( Table 2).

**Table 1:**
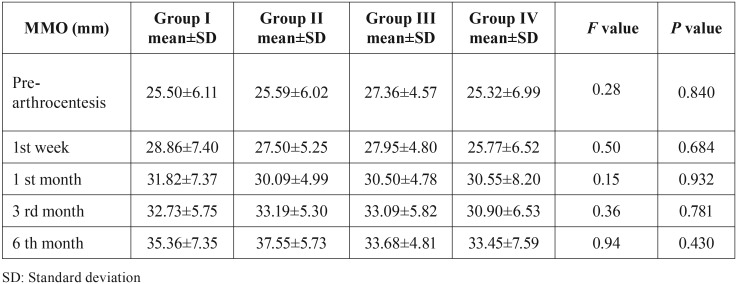
Analysis of maximal mouth opening (MMO).

**Table 2:**
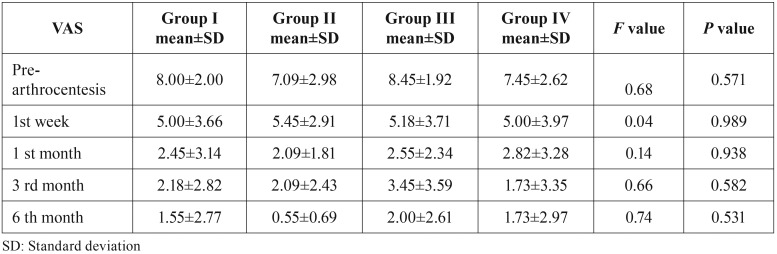
Analysis of VAS pain scores.

**Figure 1:**
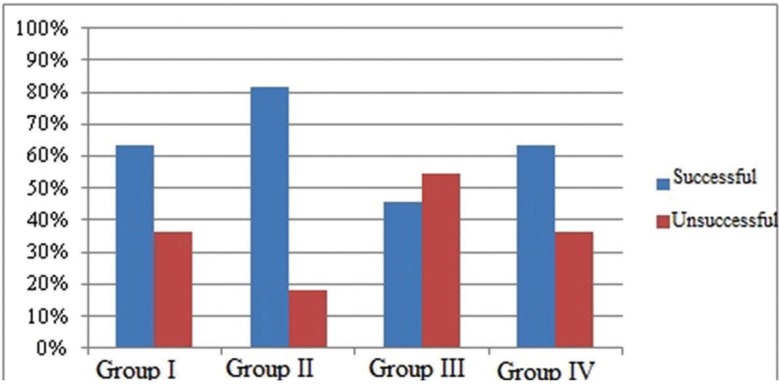
Evaluation of the groups according to treatment success.

Statistically significant differences in TMJ tenderness at palpation among groups were observed only at 1 month postoperatively (*p*<0.05). No statistically significant differences among groups were observed in the muscles of mastication regarding tenderness at palpation ( Table 3, Table 4). Overall treatment success did not very significantly among the groups (Fig. 1).

**Table 3:**
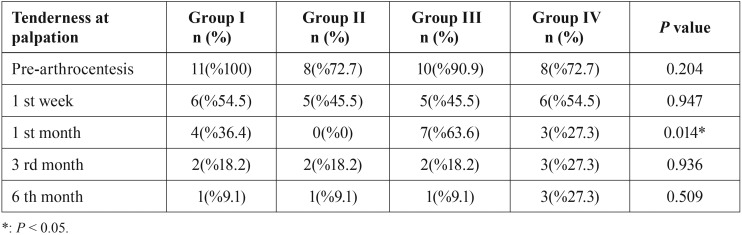
Analysis of scores of TMJ tenderness at palpation.

**Table 4:**
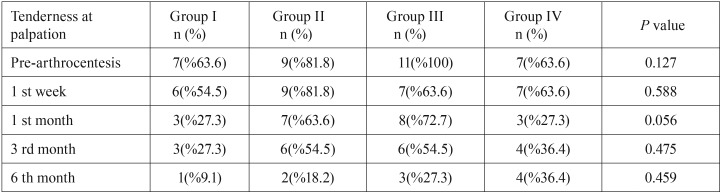
Analysis of scores of muscles of mastication tenderness at palpation.

- Radiological Findings 

**Figure 2:**
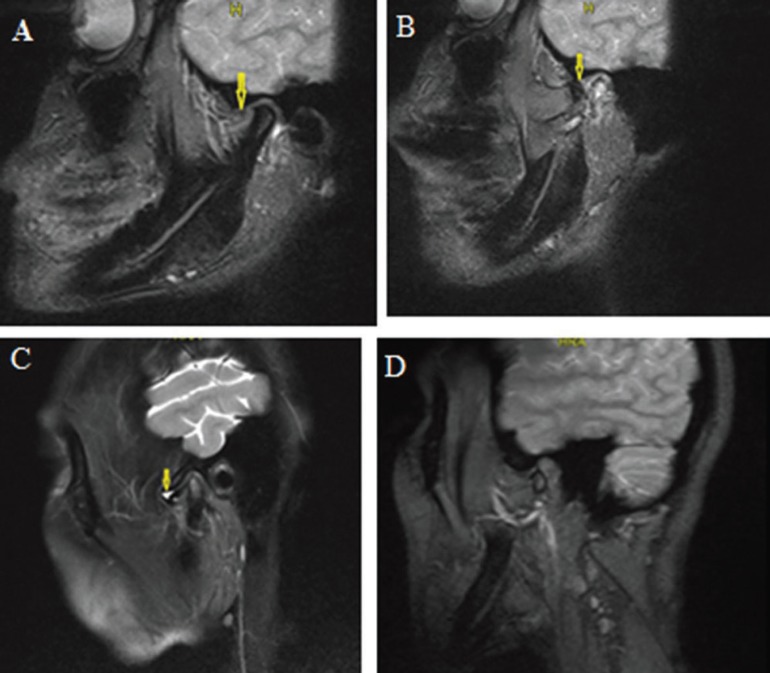
a: The disc was located anterior to the condyle prior to treatment. b: The disc was normal position after treatment. c: Image of a joint effusion prior to treatment. d: There was no effusion after treatment.

No statistically significant differences were observed in the pre- or post-treatment presence of reduction, range of motion, joint space width, or effusion levels among the groups (*p*<0.05). However, the differences between pre- and postoperative values were statistically significant for all groups (Fig. 2).

## Discussion

There are two perspectives concerning the best form of treatment for patients with non-reducing disc displacement. One involves starting with conservative treatment options to give the joint time to repair itself and adapt to derangement, and to progress to more advanced forms of treatment, such as arthrocentesis, only if no results have been obtained after a specific period of time (2). The other perspective calls for the immediate use of arthrocentesis or other simple, non-invasive technique without delay (19). Nitzan *et al.* (1) reported no treatment with physiotherapy before arthrocentesis to be unsuccessful, but found physiotherapy applied after arthrocentesis to increase treatment success further than physiotherapy alone.

Lavage of the upper joint space during arthrocentesis may be performed using either Ringer’s lactate or saline solution; however, Ringer’s lactate solution has been reported to be better tolerated by the fibrous tissue of the articular disc (20). For this reason, the present study used Ringer’s lactate solution for arthrocentesis in all patients. There is no consensus regarding the amount of solution to be used in arthrocentesis (21), and studies have been conducted using anywhere between 50-500 ml for TMJ lavage (3,22). Zardeneta *et al.* (22) reported that approximately 100 ml of solution was sufficient to remove specific proteins and proteases from the joint. The present study used 200 ml of Ringer’s Lactate solution for TMJ lavage.

Injections of a variety of drugs can be performed following TMJ arthrocentesis, with intra-articular steroids (1,8) and SH (5,6,23,24); reportedly the most common ones used following arthrocentesis performed for the treatment of non-reducing disc displacement. Intra-articular tenoxicam was also used in one study (6), and there are other studies that have not used any drug injection following arthrocentesis (19,25).

Sato *et al.* (23) reported a significant decrease in TMJ tenderness following SH injection after arthrocentesis in patients with non-reducing disc displacement, which the authors attributed to both the elimination of chemical mediators that cause pain and inflammation and SH’s ability to penetrate synovial tissue to prevent adhesion and reduce pain. Alpaslan and Alpaslan (5) reported decreases in pain and increases in MMO and lateral movement following arthrocentesis, both with and without SH, when used to treat internal derangement of the TMJ. The authors also suggested that SH provided long-term viscosity to joint surfaces and prevented the accumulation of pain-producing mediators. In a study of 28 patients with pain and internal derangement of the TMJ, Huddleston *et al.* (26) found decreases in pain and increases in MMO following arthrocentesis with or without a subsequent steroid injection, with the steroid administration having no significant effect on treatment outcomes.

There is no previous study comparing the effectiveness of SH and steroid injection following arthrocentesis in the treatment of non-reducing disc displacement; however a number of studies have compared the two alternatives in treating other TMJ diseases. Intra-articular steroid and SH injections were reported to be similarly effective in terms of increasing MMO and reducing pain in patients with TMJ internal derangement (27). Both drugs were also found to improve long-term clinical symptoms in patients with chronic arthritis of the TMJ, although SH was recommended as a better alternative because it has fewer side effects (27).

Only two studies in the literature have reported on the use of tenoxicam in conjunction with arthrocentesis of the TMJ (6,7). In one of these studies, Aktas *et al.* (6). reported an increase in MMO and a decrease in pain after arthrocentesis followed by an intra-articular injection of tenoxicam used to treat TMJ non-reducing disc displacement. In the other study, Emes *et al.* (7) Found no differences in MMO increases or pain reduction in groups injected with tenoxicam vs SH intra-articularly for the treatment of TMJ internal derangement.

The main aim in using arthrocentesis to treat diseases such as non-reducing disc displacement is to reduce pain and increase function (28). Treatment success is dependent upon effective lysis and lavage of the superior joint space rather than on any ability of arthrocentesis to reduce displacement (25). Effective lavage results in the elimination of the vacuum in the superior joint space, an increase in the viscosity of synovial fluid, and facilitation of disc and condyle translation (1). Even if the disc is not reduced, an increase in MMO and decrease in pain are established with the adaptation and repair of the joint (21). Emshoff *et al.* (25) reported a decrease in pain and an increase in mandibular movement with the application of arthrocentesis in patients with non-reducing disc displacement, but observed no change in disc position. Similarly, De Riu *et al.* (24) reported significant decreases in pain and increases in MMO in patients with internal derangement of the TMJ who received arthrocentesis followed by SH injection, but MRIs showed no change in disc position, disc morphology or bone marrow edema. In the present study, although disc reduction was observed in only 12 (27.2%) out of 44 cases, successful results were achieved in 28 (64%) out of 44 cases. This success rate is within the range of 63%-95% reported for arthrocentesis (1,19,23).

Giraddi *et al.* (8). suggested that lavage of superior joint space exerts its effects via its ability to eliminate joint effusion. In our study, the amount of effusion decreased following arthrocentesis.

Occlusal splint is an effectively therapy used for the treatment of disc displacement (29). It is suggested that splint therapy does not improve the disc position, but is strongly related to the basic decoupling of neuromuscular reflex mechanism and reduction of TMJ (30). Although previous studies compared different splint designs, there is no consensus on the selection of specific splint designs (29). Therefore, a relaxed masticatory musculature and stable physiological stress relationships in joint structures were achieved (30).

A limitation of the present study is patient population included that only patient with non-reducing disc displacement of the TMJ. Another limitation of the study is patient monitoring was only six months after artrocentesis. Additional research is required that included also patients with another TMJ diseases which may be treated with arthrocentesis and to evaluate the long-term effects on the TMJ structures from arthrocentesis and intra-articular material injections.

## Conclusions

According to the data from this study, it may be concluded that either arthrocentesis alone or arthrocentesis with methylprednisolone acetate or sodium hyaluronate or tenoxicam intra-articular injections are similarly effective and promising methods in the treatment of TMJ with non-reducing disc displacement. Considering that TMJ non-reducing disc displacement is a multifactorial complex disease, comprehensive, long-term clinical studies with a larger sample size are needed to provide a definitive recommendation regarding the best method of treatment.

## References

[1] Nitzan DW, Dolwick MF, Martinez GA (1991). Temporomandibular joint arthrocentesis: A simplified treatment for severe, limited mouth opening. J Oral Maxillofac Surg.

[2] Frost DE, Kendell BD (1999). Part II: The use of arthrocentesis for treatment of temporomandibular joint disorders. J Oral Maxillofac Surg.

[3] Tvrdy P, Heinz P, Pink R (2015). Arthrocentesis of the temporomandibular joint: A review. Biomed Pap Med Fac Univ Palacky Olomouc Czech Repub.

[4] Dolwick MF (2007). Temporomandibular joint surgery for internal derangement. Dent Clin North Am.

[5] Alpaslan GH, Alpaslan C (2001). Efficacy of temporomandibular joint arthrocentesis with and without injection of sodium hyaluronate in treatment of internal derangements. J Oral Maxillofac Surg.

[6] Aktas I, Yalcin S, Sencer S (2010). Intra-articular injection of tenoxicam following temporomandibular joint arthrocentesis: a pilot study. Int J Oral Maxillofac Surg.

[7] Emes Y, Arpinar IS, Oncu B, Aybar B, Aktas I, Al Badri N (2014). The next step in the treatment of persistent temporomandibular joint pain following arthrocentesis: A retrospective study of 18 cases. J Craniomaxillofacial Surg.

[8] Giraddi GB, Siddaraju A, Kumar B, Singh C (2012). Internal derangement of temporomandibular joint: an evaluation of effect of corticosteroid injection compared with injection of sodium hyaluronate after arthrocentesis. J Oral Maxillofac Surg.

[9] Li C, Long X, Deng M, Li J, Cai H, Meng Q (2015). Osteoarthritic changes after superior and inferior joint space injection of hyaluronic acid for the treatment of temporomandibular joint osteoarthritis with anterior disc displacement without reduction: a cone-beam computed tomographic evaluation. J Oral Maxillofac Surg.

[10] Guarda-Nardini L, Stifano M, Brombin C, Salmaso L, Manfredini D (2007). A one-year case series of arthrocentesis with hyaluronic acid injections for temporomandibular joint osteoarthritis. Oral Surg Oral Med Oral Pathol Oral Radiol Endod.

[11] Colbert ST, Curran E, O'Hanlon DM, Moran R, McCarroll M (1999). Intra-articular tenoxicam improves postoperative analgesia in knee arthroscopy. Can J Anaesth.

[12] Truelove EL, Sommers EE, LeResche L, Dworkin SF, Von Korff M (1992). Clinical diagnostic criteria for TMD. New classification permits multiple diagnoses. J Am Dent Assoc.

[13] Dworkin SF, LeResche L (1992). Research diagnostic criteria for temporomandibular disorders: review, criteria, examinations and specifications, critique. J Craniomandib Disord.

[14] Larheim TA, Westesson PL, Sano T (2001). MR grading of temporomandibular joint fluid: association with disk displacement categories, condyle marrow abnormalities and pain. Int J Oral Maxillofac Surg.

[15] Tasaki MM, Westesson PL (1993). Temporomandibular joint: diagnostic accuracy with sagittal and coronal MR imaging. Radiol.

[16] Ghanem WA (2011). Arthrocentesis and stabilizing splint are the treatment of choice for acute intermittent closed lock in patients with bruxism. J Craniomaxillofacial Surg.

[17] Okeson JP (1987). The effects of hard and soft occlusal splints on nocturnal bruxism. J Am Dent Assoc.

[18] (1992). Parameters of care for oral and maxillofacial surgery. A guide for practice, monitoring and evaluation (AAOMS Parameters of Care-92). American Association of Oral and Maxillofacial Surgeons. J Oral Maxillofac Surg.

[19] Nitzan DW, Samson B, Better H (1997). Long-term outcome of arthrocentesis for sudden-onset, persistent, severe closed lock of the temporomandibular joint. J Oral Maxillofac Surg.

[20] Shinjo H, Nakata K, Shino K, Hamada M, Nakamura N, Mae T (2002). Effect of irrigation solutions for arthroscopic surgery on intraarticular tissue: comparison in human meniscus-derived primary cell culture between lactate Ringer's solution and saline solution. J Orthop Res.

[21] Al-Belasy FA, Dolwick MF (2007). Arthrocentesis for the treatment of temporomandibular joint closed lock: a review article. Int J Oral Maxillofac Surg.

[22] Zardeneta G, Milam SB, Schmitz JP (1997). Elution of proteins by continuous temporomandibular joint arthrocentesis. J Oral Maxillofac Surg.

[23] Sato S, Ohta M, Ohki H, Kawamura H, Motegi K (1997). Effect of lavage with injection of sodium hyaluronate for patients with nonreducing disk displacement of the temporomandibular joint. Oral Surg Oral Med Oral Pathol Oral Radiol Endod.

[24] De Riu G, Stimolo M, Meloni SM, Soma D, Pisano M, Sembronio S (2013). Arthrocentesis and temporomandibular joint disorders: clinical and radiological results of a prospective study. Int J Dent.

[25] Emshoff R, Rudisch A, Bosch R, Gassner R (2000). Effect of arthrocentesis and hydraulic distension on the temporomandibular joint disk position. Oral Surg Oral Med Oral Pathol Oral Radiol Endod.

[26] Huddleston Slater JJ, Vos LM, Stroy LP, Stegenga B (2012). Randomized trial on the effectiveness of dexamethasone in TMJ arthrocentesis. J Dent Res.

[27] Kopp S, Carlsson GE, Haraldson T, Wenneberg B (1987). Long-term effect of intra-articular injections of sodium hyaluronate and corticosteroid on temporomandibular joint arthritis. J Oral Maxillofac Surg.

[28] Nitzan DW (2006). Arthrocentesis-incentives for using this minimally invasive approach for temporomandibular disorders. Oral Maxillofac Surg Clin North Am.

[29] Korkmaz YT, Altıntas NY, Korkmaz FM, Candırlı C, Coskun U, Durmuslar MC (2016). Is Hyaluronic Acid Injection Effective for the Treatment of Temporomandibular Joint Disc Displacement With Reduction?. J Oral Maxillofac Surg.

[30] Stiesch-Scholz M, Kempert J, Wolter S, Tschernitschek H, Rossbach A (2005). Comparative prospective study on splint therapy of anterior disc displacement without reduction. J Oral Rehabil.

